# Long-term implant survival in delayed breast reconstruction

**DOI:** 10.1093/bjsopen/zraf071

**Published:** 2025-07-04

**Authors:** Fredrik Brorson, Anna Paganini, Koen Simons, Anna Elander, Emma Hansson

**Affiliations:** Department of Plastic Surgery, Institute of Clinical Sciences, Sahlgrenska Academy, University of Gothenburg, Gothenburg, Sweden; Department of Plastic Surgery, Region Västra Götaland, Sahlgrenska University Hospital, Gothenburg, Sweden; Department of Plastic Surgery, Institute of Clinical Sciences, Sahlgrenska Academy, University of Gothenburg, Gothenburg, Sweden; Department of Plastic Surgery, Region Västra Götaland, Sahlgrenska University Hospital, Gothenburg, Sweden; Department of Diagnostics, Acute and Critical Care, Institute of Health and Care Sciences, Sahlgrenska Academy, University of Gothenburg, Gothenburg, Sweden; School of Public Health and Community Medicine, Sahlgrenska Academy, University of Gothenburg, Gothenburg, Sweden; Department of Plastic Surgery, Institute of Clinical Sciences, Sahlgrenska Academy, University of Gothenburg, Gothenburg, Sweden; Department of Plastic Surgery, Region Västra Götaland, Sahlgrenska University Hospital, Gothenburg, Sweden; Department of Plastic Surgery, Institute of Clinical Sciences, Sahlgrenska Academy, University of Gothenburg, Gothenburg, Sweden; Department of Plastic Surgery, Region Västra Götaland, Sahlgrenska University Hospital, Gothenburg, Sweden

**Keywords:** outcomes, explanation, implant loss, complications, revisions, breast cancer, mastectomy

## Abstract

**Background:**

The primary aim of this study was to establish the incidence of implant-related operations and revisions after delayed implant-based breast reconstruction over a 20-year period.

**Methods:**

This study is an ancillary study to the Gothenburg Breast Reconstruction Study (GoBreast; NCT03963427). The first included patient was operated on in 2003, and the last was operated on in 2011. All breast reconstructions were delayed procedures. The Kaplan–Meier method was used to estimate the time until implant loss. Log-rank tests (Mantel–Haenszel) were used for comparisons. A Cox proportional hazards model was used for multivariable analysis, and hazard ratios were estimated.

**Results:**

The study included 881 implants and 603 patients. The mean follow-up for the implants was 8.2 years. With regard to first implants, 17% had at least one unplanned procedure with implant failure. If all implants are pooled together, the 20-year implant survival rate is 57% (95% confidence interval 54 to 61%). Most implants were lost during the first 2 years, but the cumulative risk of implant loss increased steadily with time. When different surgical methods were compared, implant survival was statistically lower for direct-to-implant than for the other techniques (*P* < 0.001).

**Conclusion:**

About half of the implants in delayed breast reconstructions in this study survived for up to two decades without any additional surgery. Serial implant revisions seem more common than single implant revisions; if the first implant needed revision, there was a tendency for the second implant to also require revision.

## Teaser

This article establishes the incidence of implant-related operations and revisions after delayed implant-based breast reconstruction over a 20-year period. The study included 881 implants and 603 patients. Of the studied implants, 17% had at least one unplanned procedure with implant failure. If all implants are pooled together, the 20-year implant survival rate is 57% (95% confidence interval 54 to 61%).

## Introduction

Reconstruction of the breast after mastectomy for breast cancer may improve patients’ health-related quality of life and daily functioning^1,2^. Immediate breast reconstruction, that is, reconstruction at the time of mastectomy, is offered with increasing frequency, but delayed breast reconstruction (DBR) is still a common procedure^3^. The two principal techniques for breast reconstruction after mastectomy are autologous (using a patient's own tissues) and implant-based (using a silicone implant, with or without meshes and other adjuncts). Autologous breast reconstruction, such as free tissue transfer, is sometimes presented as the standard because it gives a natural, durable result and does not require the introduction of foreign material with long-term consequences^4^. Nonetheless, some patients are not suitable for microsurgical reconstruction for medical or other reasons, or do not feel that major surgery or an operation entailing a donor site is the best option for them. Moreover, when performed, autologous breast reconstruction requires more healthcare resources in terms of the time spent in the operating theatre and inpatient care^5^. Therefore, implant-based breast reconstruction still has a place in some situations, and knowledge of implants needs to continue to evolve to enable safe and evidence-based care.

Complications and consequences of implant-based reconstruction may result in the need for revision surgery, either as an absolute requirement or as an elective procedure due to patients’ or surgeons’ preferences. However, the distinction between these two types of revision surgery is not always clear-cut^6^. There is no consensus regarding how implant-related complications and consequences should be classified and reported^6,7^. Early reasons for implant loss include infection and wound-related complications such as necrosis, dehiscence, and implant exposure^8,9^. It is often difficult to determine the underlying cause in patients with these conditions because wound-related complications frequently lead to infection and vice versa^9^. Risk factors for these complications include a high body mass index or age, smoking, and a longer operative time^9–11^. Late complications and consequences that may warrant revision surgery and new operations include implant malposition, capsular contracture/pain, suboptimal implant size, implant rupture, breast implant-associated anaplastic large cell lymphoma (BIA-ALCL), and patient worry/breast implant illness^12,13^. There are now breast implant registries in several countries, which facilitates monitoring. However, the validity of the data from these registries depends on capture rates, and, to date, few registries have long-term data^14^.

There are limited data on the incidence of implant loss in the long term after breast reconstruction, particularly regarding DBR. The rate of at least one unanticipated operation was reported to be 29.4% (47 of 160) over a follow-up of 61 months in an Australian single-centre study^15^ including 160 DBRs and 14.4% (433 of 2093) at 48 months an Australian registry-based study^12^. A Danish prospective study^16^ reported a 10-year cumulative reoperation frequency of 35.1% (196 of 559) and a Canadian study^17^ found 0.7 (standard deviation (s.d.) 1.5) unanticipated operations per implant-based DBR, with an overall frequency of 44.0% (412 of 936) after a mean follow-up of 5 years. Further details of these studies are summarised in *Appendix S1*. In summary, the longest previous follow-up of implant-related reoperations in DBR seems to be 10 years. It appears that the cumulative incidence of implant loss increases with time.

The primary aim of the present study was to establish the incidence of implant-related operations and revisions after implant-based DBR over a 20-year period. Secondary aims were to investigate differences in these complications between different reconstruction techniques and the effects of previous revisions on implant-related operations. This information is essential for patients and surgeons to have realistic expectations about what implant-based DBR entails.

## Methods

### Study design, protocol, and ethics

This study is an ancillary study to the Gothenburg Breast Reconstruction Study (GoBreast; NCT03963427) and is reported according to the STROBE guidelines^18^.

The ancillary protocol was vetted by the Swedish Ethical Review Authority (2021-06131-02). The procedures followed were in accordance with the Declaration of Helsinki and Good Clinical Practice (GCP) guidelines. All patients were contacted before data collection, and an opt-out approach was used to obtain consent. Only those who consented to participation, data collection, and publication were included in the study.

### Setting and reconstruction protocol

The study was conducted at the Department of Plastic and Reconstructive Surgery, Sahlgrenska University Hospital (Gothenburg, Sweden). The department has a catchment area of 1.7 million inhabitants and performs approximately 350–400 breast reconstructions per year. The Swedish healthcare system is a publicly funded welfare-type system, and breast reconstructions in the private sector are performed only sporadically. Therefore, this study covers most DBRs performed in western Sweden, with a catchment area of approximately 2 million inhabitants, during the study period.

A new follow-up was performed for previously reported implant-based breast reconstructions^10,19,20^. The first included patient was operated on in 2003, and the last was operated in 2011. All breast reconstructions were delayed procedures performed at the earliest 1 year after completing oncological treatment for breast cancer. All patients fulfilled the criteria for a breast reconstruction according to the Swedish guidelines, namely a body mass index ≤ 30 kg^2^/cm, American Society of Anesthesiologists (ASA) physical status ≤ II, and abstinence from smoking six weeks before and after the operation^21^.

The surgical method chosen from 2003 to early 2009 was left to the discretion of surgeons. Implant-based reconstruction methods used in the department were a one-stage lateral thoracodorsal flap with a permanent implant (TD)^22^ (later referred to as a lateral intercostal artery perforator flap)^23^, two-stage expander reconstruction (EXP)^24^, latissimus dorsi flap reconstruction (LD) combined with a permanent implant^25^, and direct-to-implant (DTI)^20^. Patients who had undergone radiotherapy were mainly operated with an LD or a deep inferior epigastric artery perforator flap^19,26^. Between 2009 and 2011, patients who had not had radiotherapy were randomized to either the TD or EXP methods, and those who had undergone radiotherapy were randomized to either LD combined with a permanent implant or a deep inferior epigastric artery perforator flap. The surgical techniques have been described previously^19,20^. Only patients with implants and only patients with primary implants inserted at the Department of Plastic and Reconstructive Surgery, Sahlgrenska University Hospital were included in the present study. The expanders and implants used were manufactured by Allergan (2003–2007) and by Mentor (2008–2022).

### Definition of endpoints and data collection

All charts were reviewed up until 31 December 2022. A summary of the definitions of endpoints for implant loss used in this study is provided in *Table 1*. The main indication for the operation was used as a cause, regardless of intraoperative findings, because this gives the fairest picture of why decisions on a new operation are made after implant-based DBR. Intraoperative and postoperative implant-related findings and surgical details (for example, if a capsular removal was performed) were not registered, except for BIA-ALCL. All operations were treated as if the device were exchanged. The unplanned removal of a temporary tissue expander was included as a complication but not as a planned exchange to a permanent implant. In the analyses, the tissue expander and the first implant were treated as the same entity; all the implants included were permanent implants. Demographic factors (for example, radiation and patient age) were calculated per implant.

**Table 1:** Definitions of the main preoperative indications for the operation

Reason for implant death	Definition
Infection/wound dehiscence	Implant removals due to infection, wound dehiscence, and implant exposure were merged into one category because these conditions often co-exist, and it can be difficult to determine which of them is the primary cause.
Capsular contracture/pain	This cause was used when the operating surgeon stated in the medical records that this was the primary indication for the operation. However, surgeons seldom stated the grade of capsular contracture in the operation notes, and so this category also includes a number of misplaced implants without a capsular contracture. All operations performed due to pain are also included in this category.
Malplacement	This cause was used when the operating surgeon stated in the medical records that this was the primary indication for the operation and confirmed during surgery that there was no capsular contracture present.
Suspected rupture	This cause was used when an operation was performed primarily because of suspected rupture. However, this does not mean that the implant was actually found to be ruptured during the operation.
Patient-requested correction	This cause was used when the there was no sign of capsular contracture or malplacement. Examples include a wish for a change in implant size or shape.
Patient-requested removal	This cause was used when there were no medical or cosmetic reasons to remove the implant, but the patient wanted it removed anyway.
Other	This cause was used when there were other non-implant-related medical reasons to remove the implant, such as cardiac disease.

### Statistical analyses

Descriptive data are given as the mean(s.d.) or as frequencies, as applicable. The Kaplan–Meier method was used to estimate the time until implant loss. Patients were censored at the end of the study if they did not have an event before then or earlier in the event of patient death. Patient death was considered unrelated to implant survival time and not a competing risk. The log-rank test (Mantel–Haenszel) was used for comparisons. For multivariable analysis the Cox proportional hazards model was used, and hazard ratios (HRs) were estimated. Cubic B-splines with 5 d.f. each were used to model age and the calendar year of surgery. Implant side, radiotherapy, surgical method, and bilateral *versus* unilateral implant were included as dummy indices. The proportional hazards assumption (PH) was assessed using a global test at alpha = 0.050^27^. When the global test rejected the PH, the variable with the lowest *P* value against the PH was added to the set of variables, and a new model was fitted. This procedure was repeated until the global test was not rejected. This resulted in models stratifying on the calendar year of insertion (that is, the models include separate baseline hazards for each calendar year). Within-patient clustering can result in biased standard errors and erroneous *P* values. Such clustering must be due to unmeasured patient characteristics that affect the probability of implant failure. This can result in selection bias because only patients whose first implant failed can be at risk of failure of a second implant, and, by definition, patients who are more frail are more likely to experience a first implant failure. To eliminate potential bias due to within-patient clustering, the Cox proportional hazards analyses were restricted to the first implant and were repeated with both implants and restricted to left-side implant in the event of bilateral implants. The analyses are descriptive in nature and therefore do not imply causality. *P* < 0.050 was considered statistically significant. Statistical analyses were performed using R version 4.4.1.

## Results

The primary operations were performed between 2003 and 2011, with unplanned implant-related procedures occurring up until 2022. The mean follow-up period for the implants was 8.2 years (range 0 years (0 months)–19 years (230 months)). The study included 881 implants and 603 patients (*Table 2*), of whom 74 had bilateral implants, resulting in 677 first implants. Five patients declined to participate in the study, and six patients were excluded because they emigrated from Sweden during the follow-up period. At the time of the primary implant operation, patients ranged in between 28 and 78 years. Approximately half the operations (55.4%) were reconstructions performed in two stages, where a temporary tissue expander was first used and later exchanged for a permanent implant. One of the patients operated on due to suspected rupture had BIA-ALCL confirmed in the pathological anatomical investigation. This case has been reported previously^27^. In all, 152 patients were censored because they died during the study period.

**Table 2:** Demographics of the studied cohort

	Total (*n* = 881)	Implant						
		First (*n* = 677)	Second (*n* = 113)	Third (*n* = 66)	Fourth (*n* = 16)	Fifth (*n* = 5)	Sixth (*n* = 3)	Seventh (*n* = 1)
Age at implant operation (years), mean(s.d.)	53 (9.7)	53 (9.5)	54 (9.7)	56 (10.5)	52 (8.7)	56 (9.3)	64 (12.1)	73
**Radiotherapy**								
Yes	292 (33.1%)	223 (32.9%)	40 (35.4%)	24 (36%)	3 (19%)	1 (20%)	1 (33%)	0
No	559 (63.5%)	430 (63.5%)	69 (61.1%)	40 (61%)	13 (81%)	4 (80%)	2 (67%)	1 (100%)
Missing	30 (3.4%)	24 (3.5%)	4 (3.5%)	2 (3%)	0	0	0	0
**Original surgical method**								
Expander + implant	488 (55.4%)	401 (59.2%)	54 (47.8%)	27 (41%)	5 (31%)	0	1 (33%)	0
Latissimus dorsi	138 (15.7%)	99 (14.6%)	18 (15.9%)	14 (21%)	4 (25%)	2 (20%)	1 (33%)	0
Direct to implant	112 (12.7%)	71 (10.5%)	23 (20.4%)	11 (17%)	5 (31%)	2 (20%)	0	0
Permanent expander	22 (2.5%)	19 (2.8%)	2 (1.8%)	1 (2%)	0	0	0	0
Thoracodorsal	121 (13.7%)	87 (12.9%)	16 (14.2%)	13 (20%)	2 (13%)	1 (20%)	1 (33%)	1 (100%)
**Implant death**								
Total	339 (38.5%)	232 (34.3%)	75 (66.4%)	20 (30%)	7 (44%)	4 (80%)	1 (33%)	0
Infection/wound dehiscence	47 (5.3%)	28 (4.1%)	11 (9.7%)	5 (8%)	1 (6%)	1 (20%)	1 (33%)	0
Capsular contracture	192 (21.8%)	137 (20.2%)	40 (35.4%)	10 (15%)	3 (19%)	2 (40%)	0	0
Malplacement	20 (2.3%)	18 (2.7%)	2 (1.8%)	0	0	0	0	0
Suspected rupture	15 (1.7%)	9 (1.3%)	3 (2.7%)	2 (3%)	1 (6%)	0	0	0
Patient-requested correction	39 (4.4%)	28 (4.1%)	8 (7.1%)	1 (2%)	1 (6%)	1 (20%)	0	0
Patient-requested removal	1 (0.1%)	1 (0.1%)	0	0	0	0	0	0
Other	5 (0.6%)	3 (0.4%)	2 (1.8%)	0	0	0	0	0
Unknown	20 (2.3%)	8 (1.2%)	9 (8.0%)	2 (3%)	1 (6%)	0	0	0
Follow-up implants (years), mean(s.d.)	8.2 (6.5)	9.0 (6.6)	5.5 (5.4)	6.3 (5.4)	7.3 (5.8)	3.7 (4.8)	3.7 (2.9)	6.2
Time to implant loss (years), mean(s.d.)	2.7 (3.5)	2.5 (3.4)	3.5 (4.0)	1.66 (1.74)	2.1 (0.9)	1.7 (1.1)	3.2	

Values are *n* (%) unless otherwise stated.

**Fig. 1:**
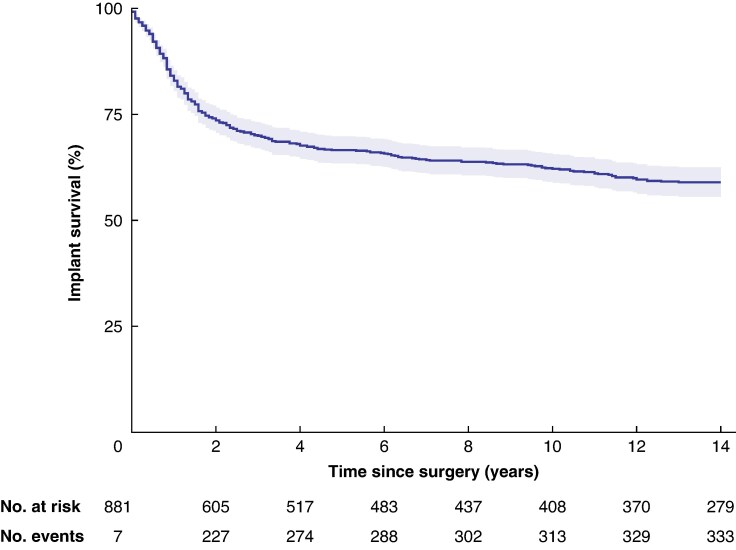
Kaplan–Meier curve showing implant survival for all implants in the study pooled together Shaded areas indicate 95% confidence intervals.

Of the studied implants, 16.7% (113 of 677) had at least one unplanned procedure with implant failure. If all implants (881) are pooled together, the 20-year implant survival rate is 57% (95% confidence interval (c.i.) 54 to 61%) (*Fig. 1*). Most implants were lost during the first 2 years, but the cumulative risk of implant loss increased steadily with time. When the analysis was restricted to the implants lost, the mean and median times to implant loss were 1.25 and 2.69 years, respectively. Restricting the analysis to the first implants that were lost, the mean and median times to implant loss were 1.1 and 2.5 years, respectively.

**Fig. 2:**
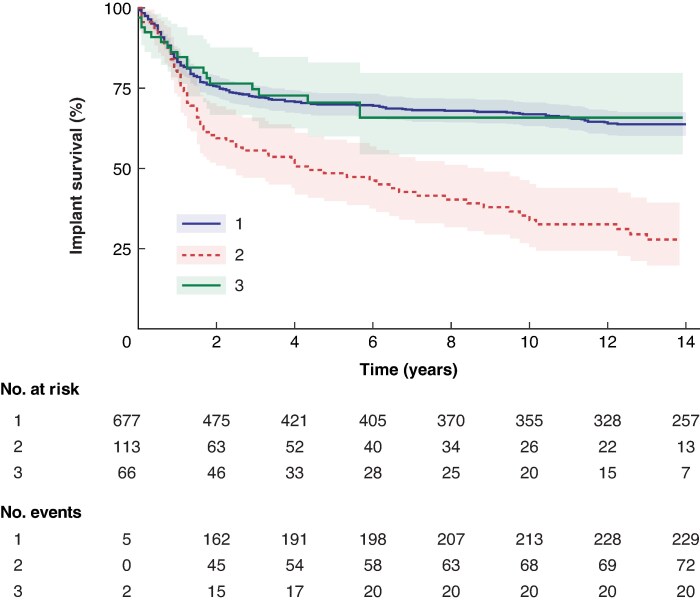
Kaplan–Meier curves for the first to third implants Shaded areas indicate 95% confidence intervals. The analysis was restricted to the first to third (1–3) implants because there were few implants in the groups with 4–7 implants (range 1–16 implants).

Implant survival time differed between the first three implants (*P* < 0.001). Specifically, implant survival was longer for the first and third implants than for the second implant (*P* < 0.001), with 10-year implant survival rates for the first, second, and third implants of 67% (95% c.i. 63 to 71%), 34% (95% c.i. 26 to 45%), and 66% (95% c.i. 54 to 80%), respectively (*Fig. 2*).

**Fig. 3:**
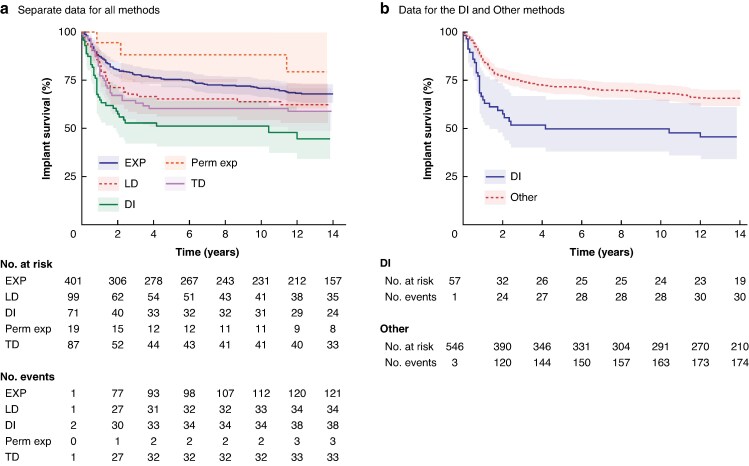
Kaplan–Meier curves of the first implant according to the primary method Shaded areas indicate 95% confidence intervals. **a** Data for all methods separately. **b** Data for the DI and Other methods. In **b**, in the event of bilateral implants, the analysis was restricted to one implant (left) per patient. DI, direct-to-implant; EXP, two-stage expander reconstruction; LD, latissimus dorsi flap; Perm exp, permanent expander in one stage; TD, thoracodorsal flap.

Among first implants (677), 13.5% of the EXP, 18.2% of the LD, 18.4% of the TD and 32.4% of the DTI first implants failed. When different surgical methods were compared, implant survival was significantly lower for DTI than for the other techniques when all implants (*P* < 0.001) were analysed, when only the first implants were analysed (*P* < 0.001), and when all other methods were grouped in the sensitivity analysis (DTI *versus* Other methods; *P* < 0.001) *Fig. 3*).

**Fig. 4:**
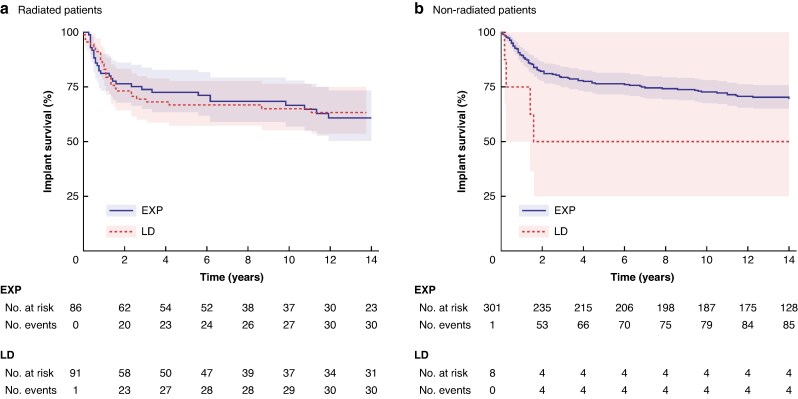
Kaplan–Meier curves for the first implant according to method Shaded areas indicate 95% confidence intervals. **a** Radiated patients. **b** Non-radiated patients. EXP, two-stage expander reconstruction; LD, latissimus dorsi flap.

In this study, 33.1% of implants (292 of 881) were placed in patients who had previously received radiotherapy to the chest wall. The most common surgical methods in the irradiated group were an LD flap (120 implants; 41.1%) and a two-stage technique with a temporary tissue expander first (108 implants; 37.0%). Among radiated patients, there was no difference in the survival of the first implant between the tissue expander and LD groups (*P* = 0.6) (*Fig. 4*).

Cox regression analysis revealed that the surgical method may be a predictor of time until the loss of the first implant, with a higher risk of loss with the DTI method (HR 2.01; 95% c.i. 1.36 to 2.99; reference method: temporary tissue expander). The risk remained when only one implant was included for patients with bilateral implants (HR 2.01; 95% c.i. 1.30 to 3.09). This HR is slightly smaller than the unadjusted HR (HR 2.2; 95% c.i. 1.54 to 3.20) (*Table 3*). Implant survival was similar for the other techniques. However, implant survival was slightly longer, albeit not statistically significant, for one-stage permanent tissue expander (HR 0.43; 95% c.i. 0.15 to 1.22) compared with a two-stage technique with a temporary tissue expander first (*Table 3*). However, these results should be interpreted with caution. To avoid rejecting the global null hypothesis of PH for all included variables, separate baselines were required for the calendar year of the implant. The *P* value against proportionality was lowest for the calendar year of the implant. In subsequently fitted models, the global test did not reject the PH; however, in some analyses, the unadjusted *P* value against the PH for the surgical method was < 0.050.

**Table 3:** Cox regression analysis for time until loss of the first implant

	Model restricted to the first implant*		Sensitivity analysis: model restricted to the first implant and one side (left)†	
	Hazard ratio	*P*	Hazard ratio	*P*
**Surgical method**				
Expander + implant	1 (Reference)	0.002	1 (Reference)	0.0076
Latissimus dorsi flap	1.34 (0.85, 2.13)		1.43 (0.89, 2.31)	
Direct to implant	2.01 (1.36, 2.99)		2.01 (1.30, 3.09)	
Permanent tissue expander	0.43 (0.15, 1.22)		0.48 (0.17, 1.37)	
Thoracodorsal flap	1.02 (0.64, 1.61)		1.06 (0.67, 1.69)	
**Other factors**				
Radiotherapy	1.01 (0.72, 1.41)	0.39	1.01 (0.70, 1.45)	0.290
Bilateral implants	1.07 (0.75, 1.52)	0.59	1.05 (0.66, 1.66)	

Values in parentheses are 95% confidence intervals. *Cox proportional hazards model with separate baselines for offending variables fitted (stratified according to the calendar year of the implant operation). Bilateral implants were added as an additive predictor. The model was adjusted for age at operation. The analysis is restricted to the first implant. The stratification did not reject the global proportional hazard assumption test (*P* = 0.124). †Cox proportional hazards model restricted to the first implant and one (left) side. The global proportional hazard assumption test was not rejected. The specific test for proportional hazard assumption of the surgical methods was rejected (*P* = 0.045).

## Discussion

Implant-based breast reconstruction is still a common procedure after breast cancer, and knowledge of the long-term surgical consequences of implants is essential to enable safe and evidence-based care. The present cross-sectional cohort study of 881 breast implants investigated the long-term incidence of implant-related operations and revisions after delayed implant-based breast reconstruction. The 20-year implant survival rate was approximately 57%. Most implants were lost during the first 2 years, but the risk of implant loss increased steadily with time. Of the studied implants, 34.3% had at least one unplanned implant-related procedure.

Long-term studies of revision surgery in delayed implant-based breast reconstructions are not common and tend to report heterogeneously defined outcomes (*Appendix S1*). A strength of the present study is that it included most patients operated on in one healthcare region with electronic medical records over the past 20 years.

The retrospective nature of this study has inherent limitations; for example, it was only possible to study the tentative reason for revision surgery, stated before surgery, and not the actual objective perioperative findings, which makes it difficult to accurately describe the factors affecting implant survival rates. Therefore, the incidence of complications such as capsular contracture and implant malfunction could not be studied; instead, the incidence of additional operations/revisions was investigated. Moreover, the clinical reason for surgery may be difficult to specify because there are often overlapping reasons for revision surgery. Moreover, reasons for revision surgery can by affected by patient or surgeon preferences and are seldom documented in a standardized fashion in medical charts. Because it was not possible to reliably study the reasons for additional surgery, a clearly defined endpoint (implant loss) was chosen to strengthen the analysis. This study can be seen as a pragmatic description of the need for additional surgery after delayed implant-based breast reconstruction in a healthcare region with a catchment area of 1.7 million inhabitants and a plastic surgery department comprising 15–20 consultant plastic surgeons performing the operations.

The pragmatic nature of this study can also be seen as a weakness, given that the choice of techniques has varied with time and with different surgeons. However, this is somewhat counterbalanced by a sample spanning many years. Nonetheless, several consecutive implant failures are uncommon and it was therefore only possible to analyse survival rates for the first three implants. The test for PH failed, which may weaken the certainty of conclusions regarding differences in implant survival between methods; however, the recent discussion^28–30^ about the usefulness of testing for proportional HRs in clinical studies suggests that the PH can be regarded as a weighted average over time, thus allowing for a higher degree of certainty.

The unit of analysis is often a matter of discussion in breast reconstruction. In this study, ‘implant’ was chosen as the unit rather than ‘patient’. This could have introduced a bias regarding patients who underwent bilateral reconstruction, because both sides could have been subjected to patient-related risks for implant revision. However, the findings that bilateral implants do not seem to be a risk factor for implant loss/revision strengthen the validity of using implant as the unit of analysis.

Implant loss/revision does not equate to reconstruction failure but can be said to indicate the minimum amount of unplanned surgery required to maintain the original reconstruction choice over time. It also indicates how predictable long-term outcomes tend to be for different methods at a group level, which is useful in clinical discussions with patients.

The findings of the present study may not be directly comparable to previous long-term follow-up studies in which other reasons for revision surgery were included. However, although the reasons for revisions and reoperations vary across different samples, a pragmatic indication of the cumulative incidence can still be obtained. In this study, the cumulative revision rate was 34% over 20 years, which is similar to previously reported incidences of 44% over 5 years^17^ and 39% over 10 years^16^. Moreover, the findings of the present study indicate that once implant failure occurs, serial implant failure is more common than having a single failure. However, third implants seem to fail less often than second implants, although these results should be interpreted with caution because this may be an effect of a shorter observation time and a smaller sample size. Nonetheless, for individuals who experience implant failure, further procedures could be more probable than not, which is important to take into consideration when choices regarding further reconstruction are made.

In the present study, DTI was a risk factor for implant loss, which is in accordance with previous findings. For example, Hvilsom *et al*.^16^ found that the 10-year incidence of reoperation with single-stage reconstructions was 52%, compared with 32% in two-stage procedures. Similarly, Finlay *et al*.^15^ reported a frequency of 61% for DTI and 28% for two-stage procedures. These previous findings corroborate the findings in the present study, namely that 34% may be a realistic 20-year incidence of implant loss in DBR and that DTI may be a risk factor for the need for unanticipated further surgery. Indeed, DTI was a popular alternative in the Department of Plastic and Reconstructive Surgery, Sahlgrenska University Hospital for only a limited period of time because it was discovered to lead to suboptimal results and more revisions, and was therefore abandoned.

In the LD group, the cumulative incidence of implant revision could be falsely high because round implants had to be used according to the GoBreast study protocol. The GoBreast study^19^ revealed that this was a suboptimal protocol, because many round implants were later exchanged for anatomical implants for cosmetic reasons. Hence, the cumulative incidence of implant revision/loss in the LD group must be interpreted with caution. The high exchange frequency from round implants to anatomical implants may also explain the much higher incidence of loss/revision in LD *versus* two-stage expander procedures in patients who had not undergone radiotherapy and a similar revision rate between these two groups among irradiated patients, as well as why radiotherapy was not a risk factor for implant loss/revision. This feature of the GoBreast protocol makes it impossible to study whether LD is a protective factor in connection with implant-based reconstruction in patients who have undergone radiotherapy and whether radiotherapy is a risk factor for implant loss/revision.

Half the implants in DBRs in this study survived for up to two decades without any additional surgery. The cumulative incidence of additional surgery was 34% over 20 years. The DTI technique seems to be a predictor of the need for non-anticipated surgery. Serial implant revisions appear more common than single implant revisions; that is, if the first implant needed revision, the second implant also tended to require revision. This is essential information to convey to patients opting for implant-based DBR. A precise classification of reasons and patient and surgeon preferences is necessary to establish the reasons for revision.

## Supplementary Material

zraf071_Supplementary_Data

## Data Availability

The participants of this study did not give written consent for their data to be shared publicly; thus, due to the sensitive nature of the research, supporting data are not available.
