# Supplementary material for: Long-term implant survival in delayed breast reconstruction
Source: BJS Open. 2025 Jul 4;9(4):zraf071. doi: 10.1093/bjsopen/zraf071 (PMC12231605; doi:10.1093/bjsopen/zraf071)
Supplement: zraf071_Supplementary_Data [file zraf071_supplementary_data.docx]

# Long-term implant survival in delayed breast reconstruction - cohort study of 881 implants

Fredrik Brorson^1,2^ MD, Anna Paganini^1,2,3^ RN, PhD, Koen Simons, PhD⁴, Anna Elander, MD, PhD^1,2^ Emma Hansson^1,2^, MD, MA, PhD

1. Department of Plastic Surgery, Institute of Clinical Sciences, The Sahlgrenska Academy, University of Gothenburg, Gothenburg, Sweden

2. Region Västra Götaland, Sahlgrenska University Hospital, Gothenburg, Department of Plastic Surgery, Sweden

3. Department of Diagnostics, Acute and Critical Care**,** Institute of Health and Care Sciences, Sahlgrenska Academy at the University of Gothenburg, University of Gothenburg, Gothenburg, Sweden

4. School of Public Health and Community Medicine, Sahlgrenska Academy at the University of Gothenburg, University of Gothenburg, Gothenburg, Sweden

**Correspondence:**

Emma Hansson, Department of Plastic Surgery, Sahlgrenska University Hospital, Röda Stråket 12, SE-413 45 Gothenburg, Sweden. Tel: +46 31 342 10 00 Fax: +46 31 82 79 03

E-mail: [emma.hansson.2@gu.se](mailto:emma.hansson.2@gu.se)

**ORCIDs**Emma Hansson 0000-0002-3218-0881

Anna Paganini 0000-0001-7043-0063

Koen Simons 0000-0002-6534-2277

**Supplementary Materials - Index**

| **Supplementary Appendixes** |  |
| --- | --- |
| Appendix S1. Summary of relevant studies | *page 2* |
|  |  |

**Supplementary Appendixes**

Appendix S1. Summary of relevant studies

| Study | Patients  Breasts | Time period for reconstructions | Follow-up | Reconstructions | Radiotherapy | Implants | Implant-related operations |
| --- | --- | --- | --- | --- | --- | --- | --- |
| Coroneos, 2019, USA ^13^  Retrospective cohort study | 99993 patients  N of breasts NR  Numbers for IBR and DBR are not reported separately. | 2007-2010 | 7 years for 27.8% of all primary reconstructions | 9942 first-time reconstructions.  Proportion of IBR/DBR NR. | NR | Mentor (53%) and Allergan (47%) | For the entire sample  Implant removal:  3.4-15.9%  Capsular contracture: 5.0-12.7% |
| Finlay, 2021, Australia ^15^  Retrospective cohort study | 390 patients  540 breasts | Jan 2005-Dec 2014 | Median 61 months (IQR 44, 88 months) | IBR: 371  DBR: 160 | Risk for revision  Implant+no radiotherapy:  OR 2.15(95% CI 1.22, 3.78)  Implant+radiotherapy:  OR 3.20 (1.31, 7.77)  Reference: pedicled flap+no radiotherapy | NR | For DBR  47/160 (29%) of the reconstruction required at least one revision |
| Hoque, 2022, Australia ^12^  Registry study (Australian Breast Device Registry) | 5152 breasts  IBR/DBR NR | 2015-2018 | Median for TE 26.5 months | TE: 3093  (71.9% therapeutic, 28.1% risk reduction)  DTI 2059 | TE: 14.3%  DTI: 6.8% | NR | Implant revision  TE 14.4% (12.5-16.7) at 48 months.  DTI: 24.4% (20.6-28.9) at 48 months |
| Hvilsom 2011 Denmark ^16^  Prospective registry study | 559 patients | 1977-2009 | Mean 4.4 (SD 3) years | DBR (n=559) | 43% (n=239) | McGahn  Mentor | Any reoperation: 35%  10-year cumulative reoperation frequency: 38.6% |
| Roberts, 2015, Canada ^17^  Retrospective cohort study | 3792 patients | 2002-2008 | Mean 5.1 years | Implant based DBR 24% (n=936) | NR | NR | Mean 0.7 (SD 1.5)  unanticipated procedures per implant based DBR.  Overall, 44% had at least one unanticipated procedure |

DBR delayed breast reconstruction DTI direct to implant IBR immediate breast reconstruction IQR intraquartal range N number of NR not reported RT radiotherapy SD standard deviation
